# Phase Boundary Mapping in ZrNiSn Half-Heusler for Enhanced Thermoelectric Performance

**DOI:** 10.34133/2020/4630948

**Published:** 2020-01-30

**Authors:** Xiaofang Li, Pengbo Yang, Yumei Wang, Zongwei Zhang, Dandan Qin, Wenhua Xue, Chen Chen, Yifang Huang, Xiaodong Xie, Xinyu Wang, Mujin Yang, Cuiping Wang, Feng Cao, Jiehe Sui, Xingjun Liu, Qian Zhang

**Affiliations:** ^1^Department of Materials Science and Engineering, Institute of Materials Genome & Big Data, Harbin Institute of Technology, Shenzhen, Guangdong 518055, China; ^2^Beijing National Laboratory for Condensed Matter Physics, Institute of Physics, Chinese Academy of Sciences, Beijing 100190, China; ^3^State Key Laboratory of Advanced Welding and Joining, Harbin Institute of Technology, Harbin, Heilongjiang 150001, China; ^4^Department of Materials Science and Engineering, Xiamen University, Xiamen, Fujian 361005, China; ^5^Department of Science, Harbin Institute of Technology, Shenzhen, Guangdong 518055, China

## Abstract

The solubility range of interstitial Ni in the ZrNi_1+*x*_Sn half-Heusler phase is a controversial issue, but it has an impact on the thermoelectric properties. In this study, two isothermal section phase diagrams of the Zr-Ni-Sn ternary system at 973 K and 1173 K were experimentally constructed based on the binary phase diagrams of Zr-Ni, Zr-Sn, and Ni-Sn. The thermodynamic equilibrium phases were obtained after a long time of heating treatment on the raw alloys prepared by levitation melting. Solubilities of *x* < 0.07 at 973 K and *x* < 0.13 at 1173 K were clearly indicated. An intermediate-Heusler phase with a partly filled Ni void was observed, which is believed to be beneficial to the lowered lattice thermal conductivity. The highest ZT value~0.71 at 973 K was obtained for ZrNi_1.11_Sn_1.04_. The phase boundary mapping provides an important instruction for the further optimization of ZrNiSn-based materials and other systems.

## 1. Introduction

Thermoelectric (TE) materials are semiconducting functional materials, which can convert heat energy directly into electricity or vice versa [1, 2]. The overall TE performance of materials is governed by the dimensionless figure-of-merit (ZT), defined as ZT = *σS*^2^*T*/(*κ*_e_ + *κ*_L_), where *σ* is the electrical conductivity, *S* the Seebeck coefficient, *κ*_e_ the electronic thermal conductivity, *κ*_L_ the lattice thermal conductivity, and *T* the absolute temperature. Good TE materials should not only have high ZTs, but also be environmentally friendly, cost-effective, stable, and strong, which arouses the interest on the promising half-Heusler alloys. Many strategies have been proven useful in enhancing the TE performance in this material system, including carrier concentration manipulating and energy band tailoring for improved power factor, and isoelectronic alloying, nanostructure constructing, and phase separation for lowered lattice thermal conductivity [3–13]. High ZTs > 1.5 have been achieved in different kinds of half-Heusler alloys, such as a high ZT~1.52 at 973 K for Ta_0.74_V_0.1_Ti_0.16_FeSb and a high ZT~1.6 at 1200 K for (Nb_1−*x*_Ta_*x*_)_0.8_Ti_0.2_FeSb (*x* = 0.36 or 0.4) [14, 15].

A typical half-Heusler crystalizes in a cubic MgAgAs-type structure (space group $F\bar{4}3m$) with the general formula *ABX*, where *A* and *B* are both transition metals and *X* is a main group element. *A* and *X* form a rock salt structure with *B* located at one of the two body diagonal positions (1/4, 1/4, 1/4). When the vacant position (3/4, 3/4, 3/4) is fulfilled by *B*, the *AB*_2_*X* full-Heusler phase (MnCu_2_Al-type, space group $Fm\bar{3}m$) forms. Lowered lattice thermal conductivity and the enhanced Seebeck coefficient are often observed in the half-Heusler alloy *ABX* composited with a small amount of the full-Heusler alloy *AB*_2_*X* with coherent boundaries [16–19]. So, a nonstoichiometric *AB*_1+*x*_*X* is generally designed. However, it is difficult to determine the excess amount of interstitial *B* atoms since there is a controversial solid solubility limit of *B* in the vacant position [20–24]. Recently, a convincing study on the solid solubility of Ni in the vacant position of TiNi_1+*x*_Sn half-Heusler has been conducted by using the phase diagram technique [25]. The narrow temperature-dependent solubility range (e.g., 0 ≤ *x* ≤ 0.06 at 1223 K in TiNi_1+*x*_Sn) was observed through phase boundary mapping on a Ti-Ni-Sn phase diagram. Many other successful examples have also been reported related to the phase diagram engineering for the enhancement of the thermoelectric properties. Tang et al. used a phase diagram approach to design the filling fraction limit of Ce in CoSb_3_ [26]. Ohno et al. controlled the doping concentration of the Zintl phase Ca_9_Zn_4+*x*_Sb_9_ by using phase boundary mapping [27].

Herein, the phase equilibria of a Zr-Ni-Sn ternary system at 973 K and 1173 K were experimentally examined. The obtained isothermal section phase diagrams clearly indicate the solubility limit of Ni in the vacant position of ZrNi_1+*x*_Sn, where *x* < 0.07 at 973 K and *x* < 0.13 at 1173 K. Samples with *x* ≤ 0.13 were selected based on the obtained phase equilibria points at 1173 K. The influence of interstitial Ni content on the microstructure and thermoelectric performance of ZrNi_1+*x*_Sn_*y*_ alloys was discussed. A maximum ZT value~0.71 was obtained at 973 K for ZrNi_1.11_Sn_1.04_ with the intermediate-Heusler phase but without extra doping. This study provides an important instruction for the further optimization of ZrNiSn half-Heusler and other material systems.

## 2. Experimental Section

### 2.1. Sample Preparation

Zirconium (Zr, 99.7%, shot), nickel (Ni, 99.98%, chunk), and tin (Sn, 99.85%, shot) were weighted according to the nominal compositions selected on the basis of the Zr-Ni, Zr-Sn, and Ni-Sn binary phase diagrams. The ternary Zr-Ni-Sn button ingots were sealed into the evacuated quartz tubes with tantalum foil wrapped after levitation melting for several times [17, 28]. The quartz tubes were annealed at 973 K for 720 hours and 1173 K for 480 hours, followed by rapidly quenching into ice water.

Compositions of ZrNi_1+*x*_Sn_*y*_ (*x* = 0.02, 0.05, 0.11, and 0.13; *y* was determined by the obtained phase diagram) were selected under the guidance of a Zr-Ni-Sn ternary isothermal section phase diagram at 1173 K. The melted ingots were placed into a stainless-steel jar with stainless-steel balls in an argon-filled glove box, and then ball milled by a high-energy ball mill (SPEX 8000M) for 2 hours. The powder was loaded into a graphite die with an inner diameter of 12.7 mm and condensed at 1173 K for 10 min with an axial pressure of 50 MPa by spark plasma sintering (SPS). The relative density of all the samples is >98%.

### 2.2. Sample Characterization

The microstructures and compositions of the annealed ingots were investigated by optical microscopy (OM, 4XC-PC), and electron probe microanalysis (EPMA, JOEL, JXA-8100). The crystal structures of sintered pellets were examined by X-ray diffraction spectra on a Rigaku D/max 2500 PC instrument with Cu K_*α*_ (*λ* = 1.5418 Å) radiation and a scanning rate of 5°min^−1^. The microstructures of sintered samples were investigated by a scanning electron microscope (SEM, Hitachi S4700) and a spherical aberration-corrected (Cs-corrected) electron microscope (JEM-ARM200F). The Seebeck coefficient (*S*) and electrical conductivity (*σ*) were simultaneously measured on a commercial apparatus (ZEM-3, Advance-Riko) from room temperature to 973 K. The temperature-dependent Hall coefficient (*R*_H_) was measured using the van-der-Pauw technique under a reversible magnetic field of 1.5 T. The Hall carrier concentration (*n*_H_) and the Hall mobility (*μ*_H_) were calculated via *n*_H_ = 1/(*eR*_H_) and *μ*_H_ = *R*_H_/*ρ*, respectively. The thermal conductivity (*κ*) was calculated using *κ* = *DαC*_p_, where *D* is the volumetric density determined by the Archimedes method, *α* is the thermal diffusivity measured using a laser flash technique (Netzsch LFA 457), and *C*_p_ is the specific heat capacity measured by a differential scanning calorimetry thermal analyzer (Netzsch DSC 404 F3). The uncertainty for the electrical conductivity is 3%, the Seebeck coefficient is 5%, and the thermal conductivity is 7% (comprising uncertainties of 4% for the thermal diffusivity, 5% for the specific heat, and 3% for the density). As a result, the combined uncertainty for the power factor is 10% and that for the ZT value is 13%. The uncertainty for the phase boundary is about 3%.

## 3. Results and Discussion

**Figure 1:**
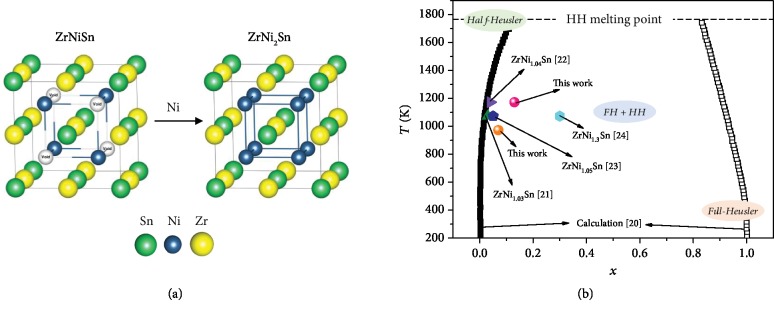
(a) Crystal structures of half-Heusler ZrNiSn and full-Heusler ZrNi_2_Sn. (b) Solubility limit of Ni in ZrNi_1+*x*_Sn half-Heusler (MgAgAs-type) [20–24].

Figure 1(a) shows the crystal structure of ZrNiSn, where Zr occupies the (1/2, 1/2, 1/2) site, the Ni (1/4, 1/4, 1/4) site, and the Sn (0, 0, 0) site, while the (3/4, 3/4, 3/4) site is vacant. When the vacant position is fulfilled by Ni, the ZrNi_2_Sn full-Heusler phase forms. To elucidate the transition process from half-Heusler to Heusler, the first-principles phase diagram calculations have been conducted [24]. As presented in Figure 1(b), there is a miscibility gap between ZrNiSn and ZrNi_2_Sn. With the increasing content of interstitial Ni, the half-Heusler alloy changes to the composite of half-Heusler with full-Heusler and at last completely changes to the full-Heusler alloy. With increasing temperature, the solubility of Ni increases in the half-Heusler alloy and decreases in the full-Heusler alloy. Since the half-Heusler phase has superior TE properties, more studies are focusing on the boundary of the half-Heusler site. We pointed out the reported experimental solubility limit in ZrNi_1+*x*_Sn, which is around 0.03 ≤ *x* ≤ 0.05 at 1100-1200 K [20–22]. More differently, Romaka et al. found this solubility up to *x* = 0.3 at 1100 K [23]. By using the phase diagram technique, the boundary seats approximately at *x* < 0.07 at 973 K and *x* < 0.13 at 1173 K in this work.

**Figure 2:**
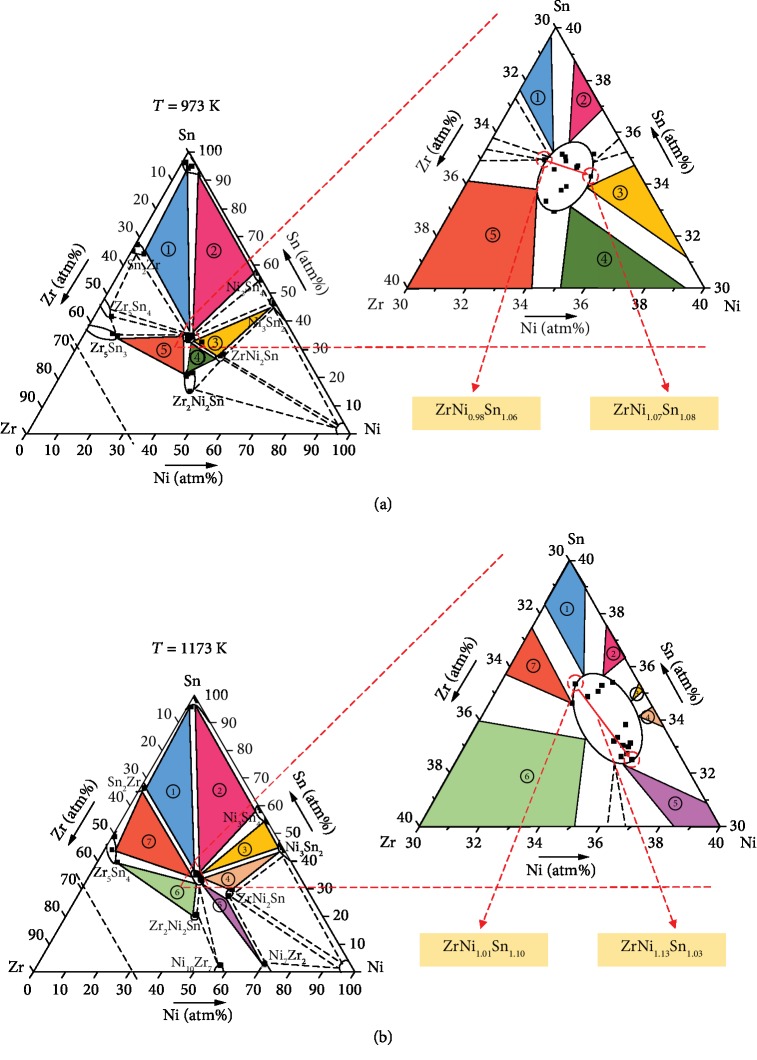
Isothermal section diagrams and magnified area of the Zr-Ni-Sn ternary system (a) at 973 K and (b) at 1173 K.

To obtain the ternary isothermal section phase diagram of the Zr-Ni-Sn system, we selected the nominal compositions according to the binary phase diagram of Zr-Ni, Zr-Sn, and Ni-Sn at 973 K and 1173 K, respectively (shown in [Supplementary-material supplementary-material-1] and [Supplementary-material supplementary-material-1]; see details for the construction of the isothermal section diagram in Supplementary Information). Based on the phase equilibrium data, the phase mapping boundary of the Zr-Ni-Sn ternary system was confirmed.  Figure 2(a) shows the complete isothermal section diagram and its central magnified area of Zr-Ni-Sn at 973 K. At this temperature, several compounds such as Sn_2_Zr, Ni_3_Sn_4_, Ni_3_Sn_2_, ZrNi_2_Sn, Zr_2_Ni_2_Sn, and Zr_5_Sn_3_ were detected, indicating five three-phase regions in this phase diagram marked by different colors. Amplifying the center area, a single phase zone was observed for ZrNi_1+*x*_Sn (wrapped in an oval), showing that the leftmost component is ZrNi_0.98_Sn_1.06_ and the rightmost component is ZrNi_1.07_Sn_1.08_. When the temperature increased to 1173 K, the number of the three-phase regions increased to seven, including (Sn+Sn_2_Zr+ZrNiSn), (Sn+Ni_3_Sn_4_+ZrNiSn), (Ni_3_Sn_4_+Ni_3_Sn_2_+ZrNiSn), (Ni_3_Sn_2_+ZrNi_2_Sn+ZrNiSn), (Ni_7_Zr_2_+ZrNi_2_Sn+ZrNiSn), (Zr_5_Sn_4_+Zr_2_Ni_2_Sn+ZrNiSn), and (Zr_5_Sn_4_+Sn_2_Zr+ZrNiSn), as shown in Figure 2(b). The magnified central area reveals that the leftmost component is ZrNi_1.01_Sn_1.10_ and the rightmost component is ZrNi_1.13_Sn_1.03._ The solubility limit of Ni in ZrNi_1+*x*_Sn half-Heusler increased from *x* = 0.07 at 973 K to *x* = 0.13 at 1173 K. You can find the typical phase compositions detected by back-scattered electron microscopy in Tables [Supplementary-material supplementary-material-1] and [Supplementary-material supplementary-material-1] and Figures [Supplementary-material supplementary-material-1] and [Supplementary-material supplementary-material-1]. Since we only focus on the ZrNi_1+*x*_Sn single-phase region, a series of alloy points around the center point were selected for the determination of some of the three-phase zones. The triangles enclosed by the black dotted line and the blank parts are trivial.

**Figure 3:**
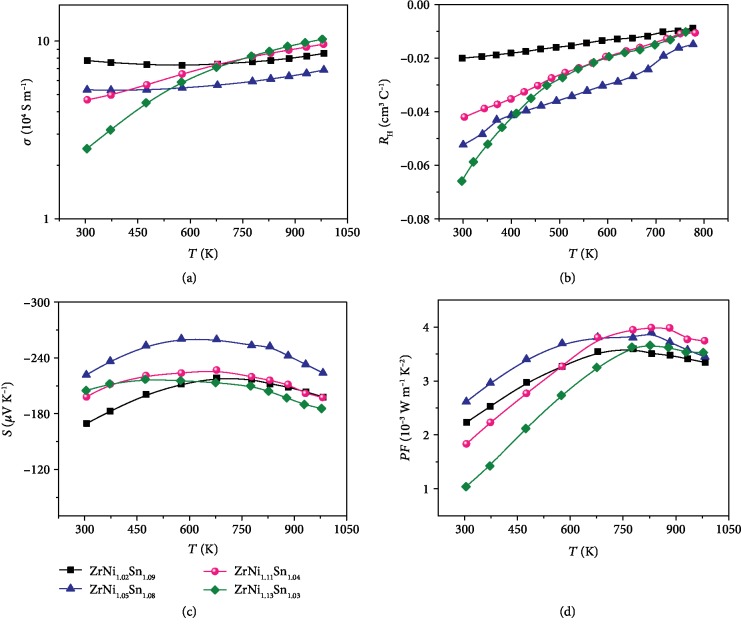
Temperature-dependent (a) electrical conductivity, (b) Hall coefficient, (c) Seebeck coefficient, and (d) power factor for ZrNi_1+*x*_Sn_*y*_ (*x* = 0.02, 0.05, 0.11, and 0.13; *y* is determined by the isothermal section phase diagram at 1173 K).

According to the isothermal section diagram at 1173 K (see Figure 2(b)), we selected four compositions within the single phase region to study the effect of interstitial Ni content on the TE performance. Samples with *x* < 0.13 show a single phase without an additional impurity peak, and the lattice constant increased slightly with increasing Ni content (see [Supplementary-material supplementary-material-1]), indicating that the excess Ni atom may occupy the interstitial void in the ZrNiSn half-Heusler matrix. The full-Heusler phase emerged in the sample with *x* = 0.13. So it is safe to determine the solubility limit as *x* < 0.13, considering the experimental error around the boundary.  Figure 3 presents the temperature dependence of (a) the electrical conductivity, (b) the Hall coefficient, (c) the Seebeck coefficient, and (d) the power factor for ZrNi_1+*x*_Sn_*y*_ (*x* = 0.02, 0.05, 0.11, and 0.13; *y* was determined by the isothermal section phase diagram at 1173 K). The electrical conductivity of Ni self-doping samples increased with increasing temperature, exhibiting typical semiconductor behavior. With the increase of the Ni content, the room temperature electrical conductivity decreased, which is due to the decreased carrier density resulting from the filtering of the low energy electrons at the HH/IH/FH interfaces [18, 29]. It has been reported that when Ni is self-doped, an impurity level will exist within the band gap, leading to the temperature-dependent carrier concentration, which is consistent with the data shown in Figure 3(b) [30–32]. The Seebeck coefficient increased first and then decreased when *x* > 0.05, and the negative value indicates an *n*-type conductive behavior, being consistent with the Hall measurement. The maximum power factor of ~4000 *μ*W m^−1^ K^−2^ was obtained at 830 K for the sample with *x* = 0.11.

**Figure 4:**
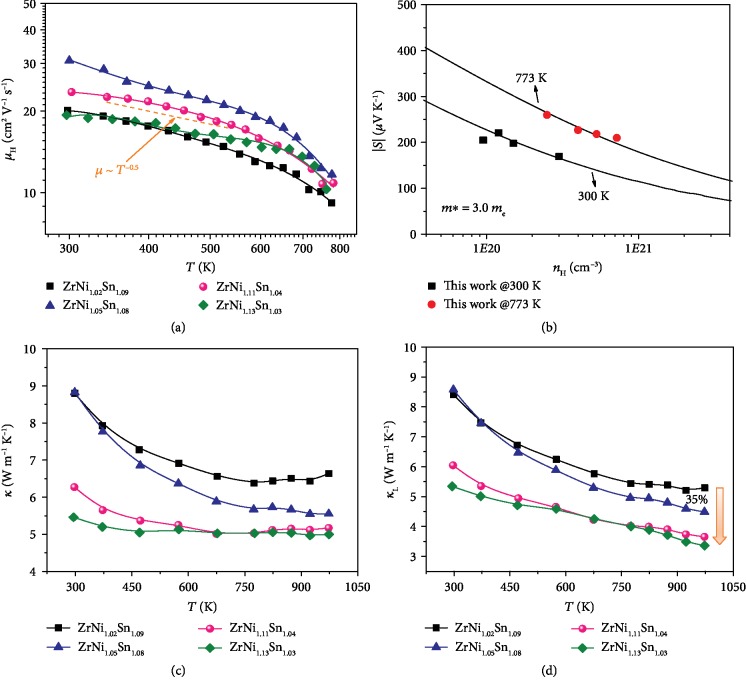
(a) The temperature-dependent Hall mobility. (b) The Seebeck coefficient as a function of the Hall carrier concentration at 300 K and 773 K. The solid lines are calculated considering acoustic phonon and alloying scattering based on the SKB model. Temperature-dependent (c) total thermal conductivity and (d) lattice thermal conductivity for ZrNi_1+*x*_Sn_*y*_ (*x* = 0.02, 0.05, 0.11, and 0.13; *y* was determined by the obtained phase diagram).

Figure 4(a) shows the temperature-dependent carrier mobility, indicating the dominated alloying scattering at a temperature lower than 650 K and acoustic phonon scattering at a higher temperature, which is consistent with previous reports [30, 33]. The Pisarenko plot of *S* versus *n*_H_ at different temperatures is presented in Figure 4(b), which is well fitted by the line calculated based on the single-Kane-band (SKB) model in view of acoustic phonon and alloying scattering [30]. The effective mass *m*^∗^ = 3.0 *m*_e_ was used in this calculation, and more details can be found in the Supplementary Information. Figures 4(c) and 4(d) present the temperature dependence of the thermal properties of the selected samples. The lattice thermal conductivity was ascertained by subtracting the electronic contribution from the total, which can be estimated by *κ*_L_ = *κ* − *LσT*, where *L* is the Lorenze number (see details in the Supplementary Information). As the Ni content rises, the room-temperature lattice thermal conductivity is reduced from 8.4 W m^−1^ K^−1^ to 5.3 W m^−1^ K^−1^, which is lower than those of the reported values of ~11.4 W m^−1^ K^−1^ for ZrNiSn and ~6 W m^−1^ K^−1^ for ZrNi_1.10_Sn with a high density of full-Huesler precipitates [34, 35]. Especially, this value is lower than those of the alloyed samples at ~13 W m^−1^ K^−1^ for Zr_0.9_Ti_0.1_NiSn and ~6 W m^−1^ K^−1^ for Zr_0.7_Ti_0.3_Ni_1.03_Sn [20, 29]. The lowest lattice thermal conductivity is ~3.4 W m^−1^ K^−1^ at 973 K for ZrNi_1.13_Sn_1.03_, suggesting the effective medium-to-high frequency phonon scattering [16, 36, 37].

**Figure 5:**
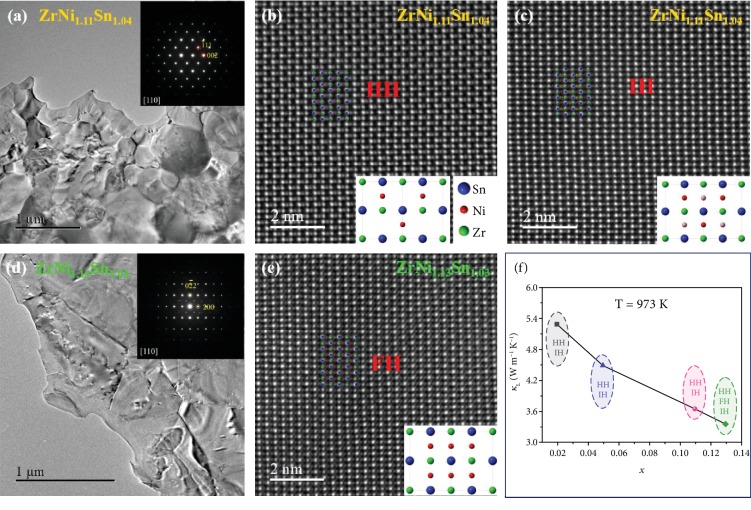
Low-magnification TEM images of (a) ZrNi_1.11_Sn_1.04_ and (d) ZrNi_1.13_Sn_1.03_; inserted are the SAED patterns along the [110] direction. HAADF-STEM images along the [110] direction of (b) ZrNi_1.11_Sn_1.04_ matched with the structure of the half-Heusler (HH) and (c) the intermediate-Heusler (IH) and (e) ZrNi_1.13_Sn_1.03_ matched with the structure of the full-Heusler (FH); (f) lattice thermal conductivity as a function of interstitial Ni content at 973 K.

The microstructures of ZrNi_1.11_Sn_1.04_ and ZrNi_1.13_Sn_1.03_ were investigated using transmission electron microscopy (TEM) and high-angle annular-dark-field scanning transmission electron microscopy (HAADF-STEM). The low-magnification TEM images show that the grain size is at microscale and the selected area electron diffraction pattern along the direction [110] is inserted (see Figures 5(a) and 5(d)), corresponding to the MgAgAs structure and the MnCu_2_Al structure, respectively. Figures 5(b) and 5(c) show the HAADF-STEM images viewed along the [110] zone axes of ZrNi_1.11_Sn_1.04_. There are two phases existing in the ZrNi_1.11_Sn_1.04_ sample. One is the typical half-Heusler (HH) with the obvious characteristic that half sites are not occupied by Ni atoms (see Figure 5(b)). The other one is demonstrated in Figure 5(c), clearly showing that slight bright spots occupy the half sites, indicating the partial Ni occupation, which could be named as intermediate-Heusler (IH) [38]. The crystal structure of the half-Heusler and the intermediate-Heusler is the same. In the sample with *x* = 0.13, besides the first two phases, the full-Heusler (FH) phase also appears (showing in Figure 5(e)). Intuitively, all Ni atoms have the same brightness and uniformly occupy the half sites. This result is consistent with the analysis of XRD (see [Supplementary-material supplementary-material-1]). In Figure 5(f), the lattice thermal conductivity versus the Ni solubility is displayed. It is generally believed that the presence of FH reduces the lattice thermal conductivity, which is also confirmed in this study (green symbols) [29, 39]. However, we should pay more attention to the interstitial Ni in the IH phase before the appearance of the FH phase (red symbols), which is significant for the decrease of the lattice thermal conductivity.

**Figure 6:**
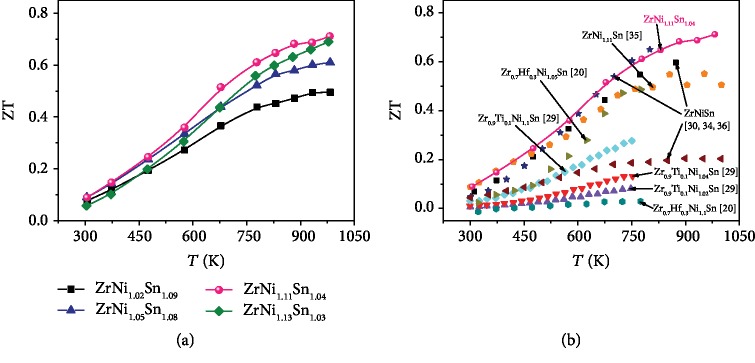
Temperature-dependent ZT for (a) ZrNi_1+*x*_Sn_*y*_ (*x* = 0.02, 0.05, 0.11, and 0.13; *y* is determined by the isothermal section phase diagram at 1173 K). (b) Comparison of ZT values for ZrNiSn-based samples [20, 29, 30, 34–36].

The ZT values of all the Ni self-doped samples are shown in Figure 6(a). With the increasing content of interstitial Ni, the ZT value increased first and decreased when the full-Heusler phase came out. The highest ZT is ~0.71 at 973 K for ZrNi_1.11_Sn_1.04_, higher than those of the other Ni self-doped ZrNi_1+*x*_Sn (see Figure 6(b)) [20, 29, 35]. In fact, the sample with the full-Heusler phase (e.g., *x* = 0.13) also has a higher ZT than that of the sample with a low concentration of interstitial Ni (e.g., *x* = 0.02). Benefiting from the precise composition determination based on the phase boundary mapping, we decreased the thermal conductivity and increased the electrical performance, leading to an enhanced ZT value, which can be applied to other material systems.

## 4. Conclusions

In summary, we constructed two isothermal section phase diagrams of the Zr-Ni-Sn ternary system at 973 K and 1173 K based on thermodynamic equilibrium, and the limited solubility of Ni in ZrNi_1+*x*_Sn_*y*_ was revealed, which is less than 0.07 at 973 K and 0.13 at 1173 K. Four samples in the 1173 K homogeneity region with different Ni contents were prepared for the exploration of the microstructure and thermoelectric performance. An enhanced ZT value~0.71 at 973 K for ZrNi_1.11_Sn_1.04_ was obtained due to the lowered lattice thermal conductivity contributed by the interstitial Ni. More importantly, interstitial atomic solid solubility also exists in other materials, and the phase diagram strategy of solid solubility determination is also applicable to other materials for improving thermoelectric properties within a wide optimization range.

## Supplementary Materials

Supplementary Materials Figure S1: (a) XRD patterns and (b) lattice constants for ZrNi_1+*x*_Sn_*y*_ (*x* = 0.02, 0.05, 0.11, and 0.13; *y* is determined by the isothermal section phase diagram at 1173 K). Figure S2: (a) Ni-Sn, (b) Ni-Zr, and (c) Zr-Sn binary phase diagrams [1–3]. Table S1: the nominal compositions and equilibrium compositions of the Zr-Ni-Sn ternary system at 973 K determined by EPMA. Table S2: the nominal compositions and equilibrium compositions of the Zr-Ni-Sn ternary system at 1173 K determined by EPMA. Figure S3: the back-scattered electron images of several typical phase compositions obtained after annealing at 973 K for 30 days. The nominal composition is presented below each image. Figure S4: the back-scattered electron images of several typical phase compositions obtained after annealing at 1173 K for 20 days. The nominal composition is presented below each image. Figure S5: temperature-dependent specific heat capacity Cp for ZrNi_1.02_Sn_1.09_.
