# Supplementary material for: Phase Boundary Mapping in ZrNiSn Half-Heusler for Enhanced Thermoelectric Performance
Source: Research (Wash D C). 2020 Jan 30;2020:4630948. doi: 10.34133/2020/4630948 (PMC7013278; doi:10.34133/2020/4630948)
Supplement: Supplementary Materials — Figure S1: (a) XRD patterns and (b) lattice constants for ZrNi1+xSny (x = 0.02, 0.05, 0.11, and 0.13; y is determined by the isothermal section phase diagram at 1173 K). Figure S2: (a) Ni-Sn, (b) Ni-Zr, and (c) Zr-Sn binary phase diagrams [1–3]. Table S1: the nominal compositions and equilibrium compositions of the Zr-Ni-Sn ternary system at 973 K determined by EPMA. Table S2: the nominal compositions and equilibrium compositions of the Zr-Ni-Sn ternary system at 1173 K determined by EPMA. Figure S3: the back-scattered electron images of several typical phase compositions obtained after annealing at 973 K for 30 days. The nominal composition is presented below each image. Figure S4: the back-scattered electron images of several typical phase compositions obtained after annealing at 1173 K for 20 days. The nominal composition is presented below each image. Figure S5: temperature-dependent specific heat capacity Cp for ZrNi1.02Sn1.09. [file 4630948.f1.docx]

**Phase Boundary Mapping in ZrNiSn Half-Heusler for Enhanced Thermoelectric Performance**

Xiaofang Li^a#^, Pengbo Yang^a#^, Yumei Wang^b^, Zongwei Zhang^a^, Dandan Qin^c^, Wenhua Xue^b^, Chen Chen^a^, Yifang Huang^a^, Xiaodong Xie^a^, Xinyu Wang^a^, Mujin Yang^a^, Cuiping Wang^d^, Feng Cao^e^, Jiehe Sui^c*^, Xingjun Liu^a, c*^, Qian Zhang^a*^

*^a^Department of Materials Science and Engineering, and Institute of Materials Genome & Big Data, Harbin Institute of Technology, Shenzhen, Guangdong 518055, P.R. China, E-mail:* [zhangqf@hit.edu.cn](mailto:zhangqf@hit.edu.cn), xjliu@hit.edu.cn

*^b^Beijing National Laboratory for Condensed Matter Physics, Institute of Physics, Chinese Academy of Sciences, Beijing 100190, P.R. China*

*^c^State Key Laboratory of Advanced Welding and Joining, Harbin Institute of Technology, Harbin, Heilongjiang 150001, P.R. China, E-mail:* [suijiehe@hit.edu.cn](mailto:zhangqf@hit.edu.cn)

*^d^Department of Materials Science and Engineering, Xiamen University, Xiamen, Fujian 361005, P.R. China*

*^e^Department of Science, Harbin Institute of Technology, Shenzhen, Guangdong 518055, P.R. China*

# Equal contributors

**I. XRD patterns and lattice constants**

**Fig. S1** (a) XRD patterns, (b) lattice constants for ZrNi_1+_*_x_*Sn*_y_* (*x* = 0.02, 0.05, 0.11, and 0.13, *y* is determined by the isothermal section phase diagram at 1173 K).

**II. Isothermal section phase diagram of the Zr-Ni-Sn ternary system**

There are several steps for constructing the isothermal section phase diagram (973 K or 1173 K) of the Zr-Ni-Sn ternary system. First, all the related phase diagram information, including the corresponding sub-systems (Zr-Ni, Zr-Sn, and Ni-Sn), the known binary/ternary compounds (like Ni_3_Sn_2_, *etc*.), and their binary solubility, *etc*. are collected and analyzed. Based on this, a possible ternary phase (like ZrNiSn or ZrNi_2_Sn) relationship could be expected. And then, several ternary alloy compositions (like Zr_40_Ni_30_Sn_30_ alloy, *etc*., as listed in Tab. S1, S2) were designed, prepared by arc-melting, and heat-treated at 973 K or 1173 K for a long time for equilibrium. Third, these equilibrium samples were quenched into ice-water and analyzed by EPMA and XRD for the phase composition, relationship, and crystal structure identification. Based on the obtained information, the isothermal section of the Zr-Ni-Sn ternary system at 973 K or 1173 K is established as presented in Fig. 2. In the meantime, the phase boundary was revealed.

**1. Ni-Sn, Ni-Zr, and Zr-Sn binary phase diagrams**


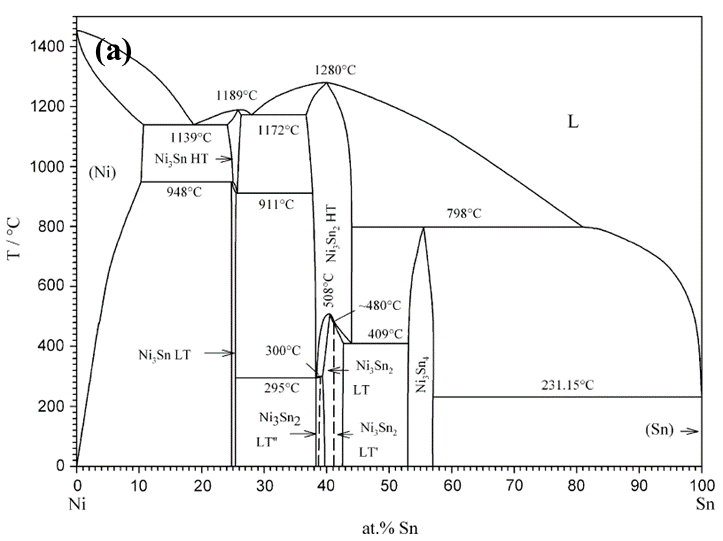


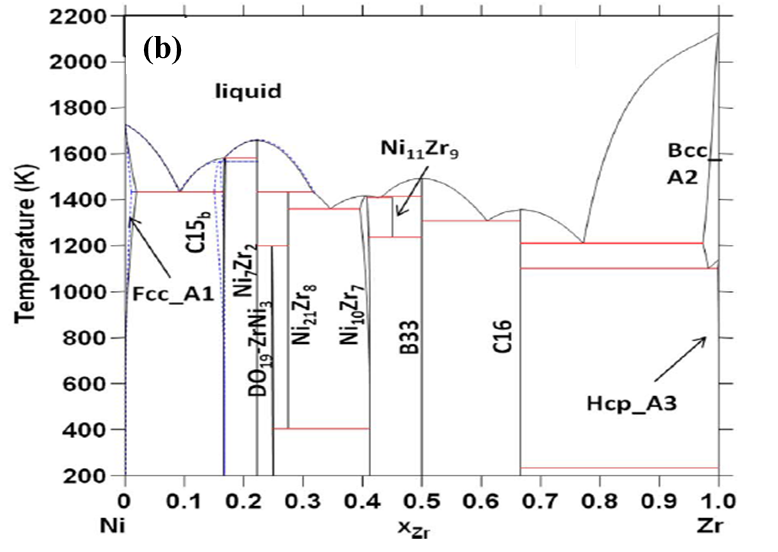


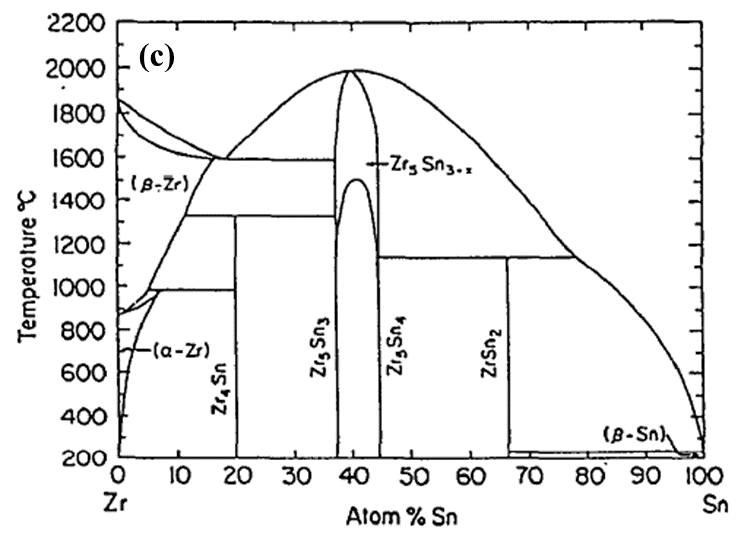


**Fig. S2** (a) Ni-Sn, (b) Ni-Zr, and (c) Zr-Sn binary phase diagram.^1-3^

1. **EPMA data for Zr-Ni-Sn ternary system at different temperature**

**Tab. S1** The nominal compositions and equilibrium compositions of Zr-Ni-Sn ternary system at 973 K determined by EPMA


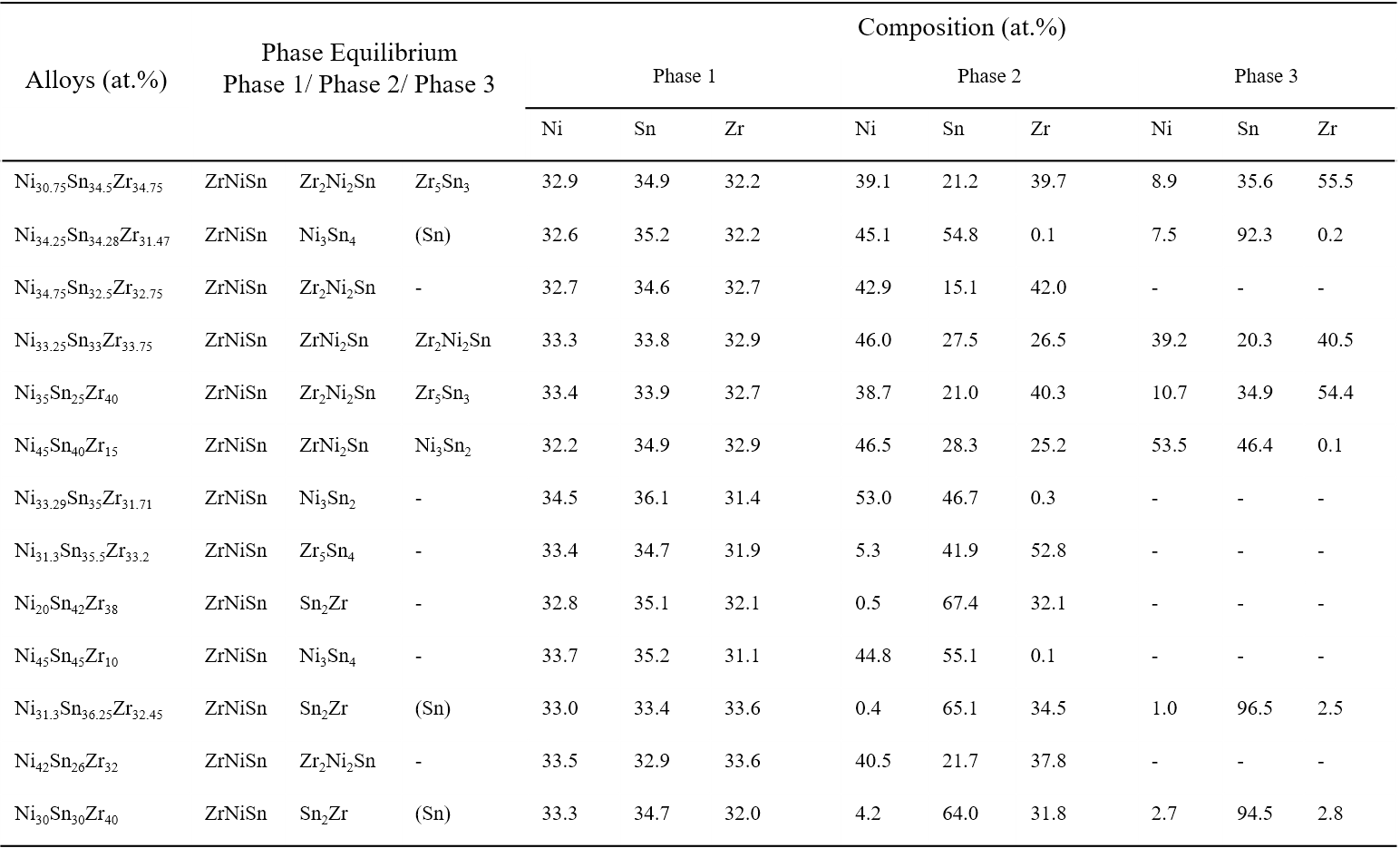


**Tab. S2** The nominal compositions and equilibrium compositions of Zr-Ni-Sn ternary system at 1173 K determined by EPMA


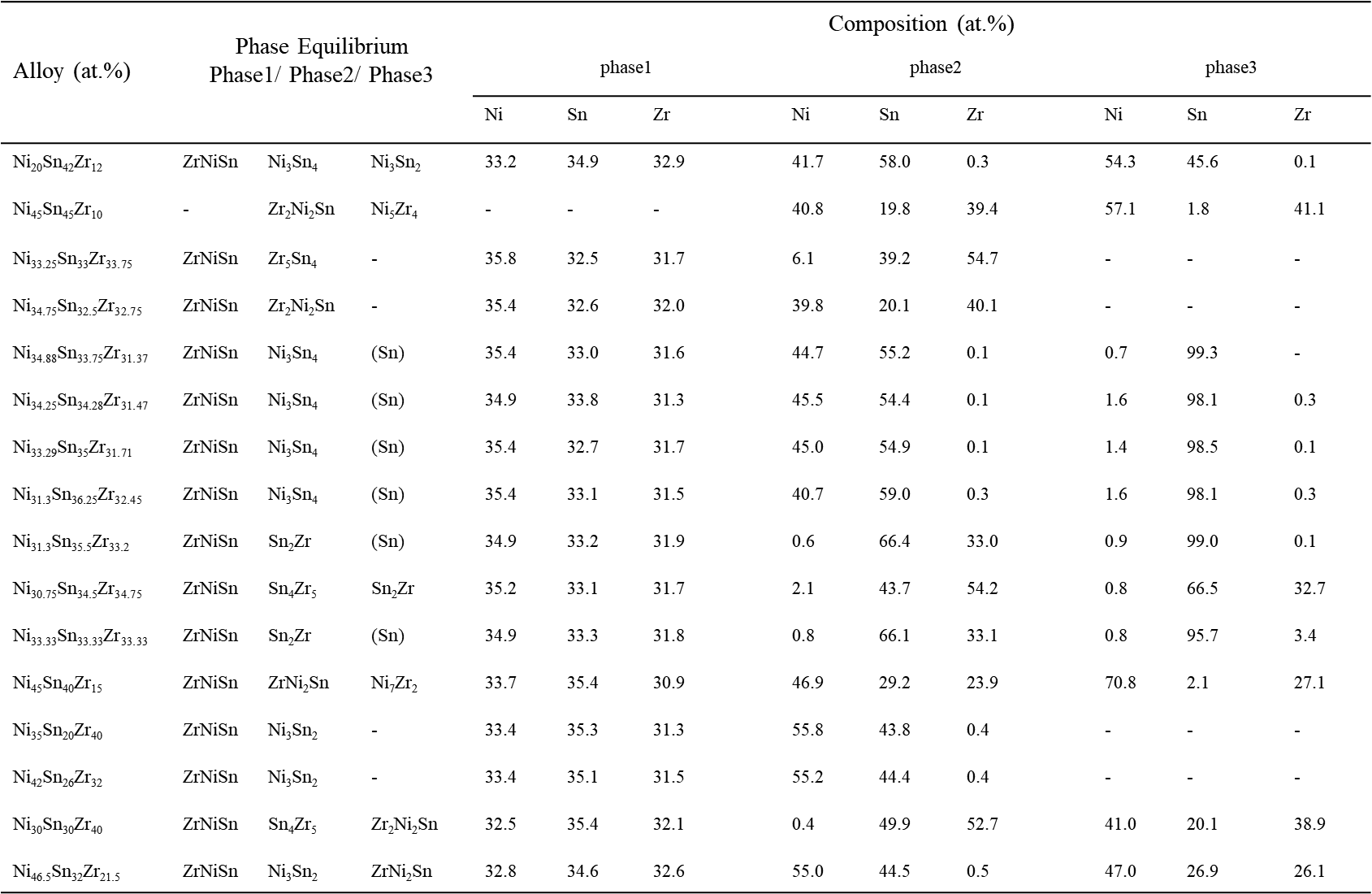


**3. Back-scattered electron images of typical phase compositions**


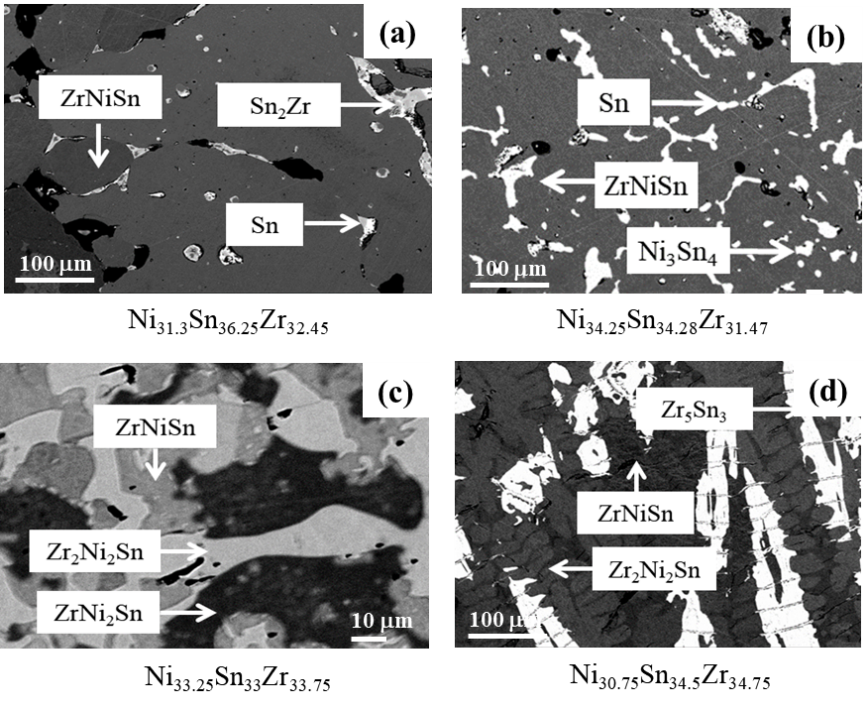


**Fig. S3** The back-scattered electron images of several typical phase compositions obtained after annealing at 973 K for 30 days. The nominal composition is presented below each image.


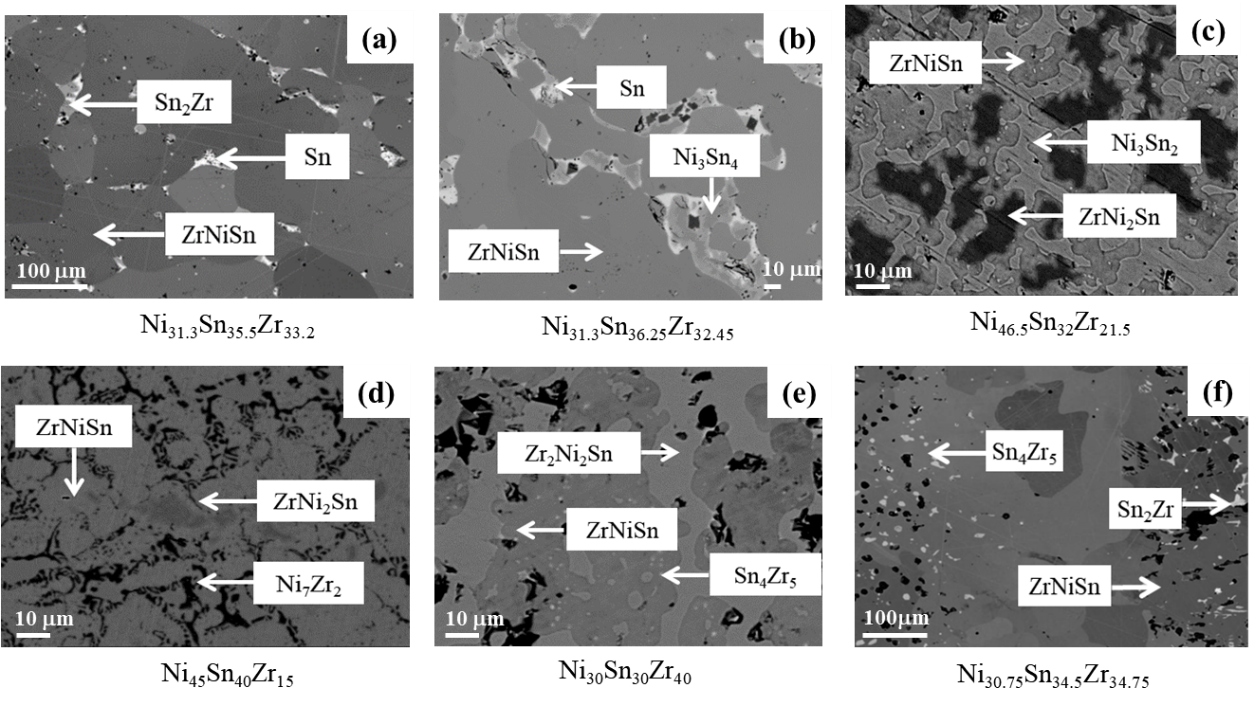


**Fig. S4** The back-scattered electron images of several typical phase compositions obtained after annealing at 1173 K for 20 days. The nominal composition is presented below each image.

**III. Specific heat capacity**


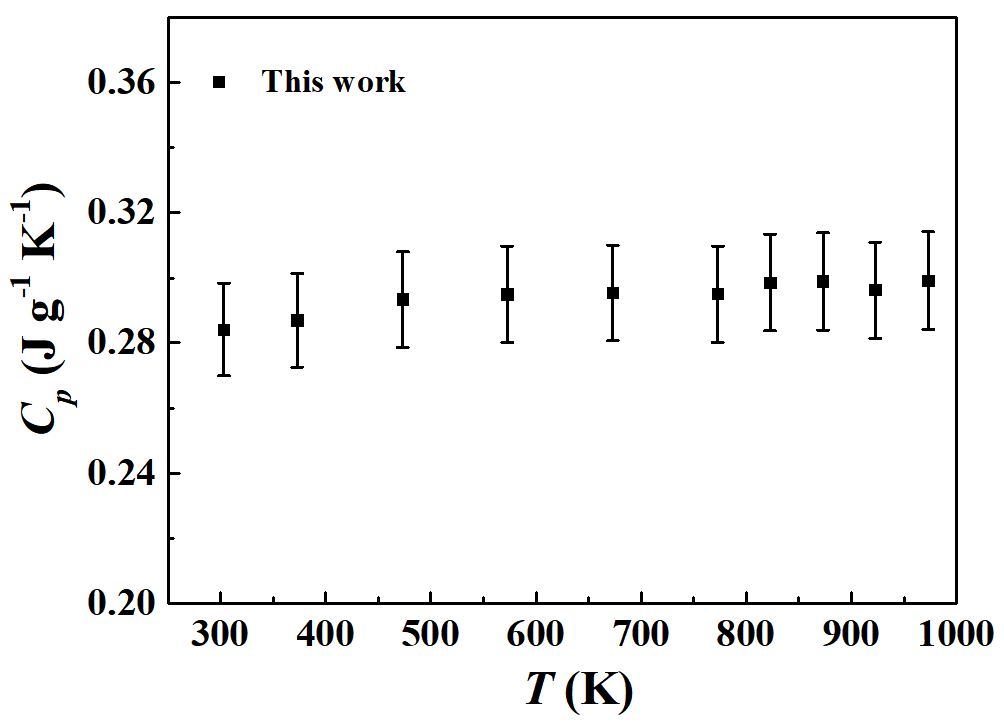


**Fig. S5** Temperature-dependent specific heat capacity *C_p_* for ZrNi_1.02_Sn_1.09_.

**IV. Single-Kane-band model details**

In this paper, acoustic phonon scattering and alloy scattering are considered to be the main scattering mechanisms, and the total relaxation time determined by Matthiessen’s rule:

$\tau_{total}^{-1}\text{=}\tau_{ac}^{-1}+\tau_{alloy}^{-1}$.

The relaxation time for acoustic phonon scattering based on deformation potential theory can be expressed as:

$$\varepsilon\text{=}\frac{E}{k_{B}T}$$

Here, *k*_B_ is the Boltzmann constant, $m_{d}^{*}$ is the total density of state effective mass, *N*_V_ is the band degeneracy and its value is 3 here, $\nu_{l}$is the longitudinal velocity, $\rho$ is the density, $E_{def}$ is the deformation potential ~5 eV, and $\alpha\text{=}k_{B}T/E_{\mathrm{g}}$, where $E_{\mathrm{g}}$ is the energy gap at X point, $\varepsilon$ is the reduced carrier energy.

The relaxation time for alloy scattering can be expressed as:

Here, $\Omega$ is the volume per atom, *x* is the concentration ratio of the alloy atom, $E_{al}$ is the alloy scattering potential ~1 eV, and $m_{b}^{*}$ is the density-of-state effective mass for a single valley defined as $m_{d}^{*}=N_{\text{V}}^{2/3}m_{b}^{*}$.

The generalized Fermi integral ${}^{n}F_{l}^{m}$is defined by

$${}^{n}F_{l}^{m}=\int_{0}^{\infty} (-\frac{\partial f}{\partial\varepsilon})\varepsilon^{n}(\varepsilon+\alpha\varepsilon^{2})^{m}(1+2\alpha\varepsilon)^{l}d\varepsilon$$

The transport parameters can be expressed using SKB model. The Seebeck coefficient *S*:

$$S=\frac{k_{B}}{e}(\frac{\int_{0}^{\infty} (-\frac{\partial f}{\partial\varepsilon})\tau_{total}\varepsilon^{5/2}(1+\alpha\varepsilon)^{3/2}(1+2\alpha\varepsilon)^{-1}d\varepsilon}{\int_{0}^{\infty} (-\frac{\partial f}{\partial\varepsilon})\tau_{total}\varepsilon^{3/2}(1+\alpha\varepsilon)^{3/2}(1+2\alpha\varepsilon)^{-1}d\varepsilon}-\eta)$$

The Lorenz number *L* is given by:

$L=(\frac{k_{B}}{e})^{2}[\frac{\int_{0}^{\infty} (-\frac{\partial f}{\partial\varepsilon})\tau_{total}\varepsilon^{7/2}(1+\alpha\varepsilon)^{3/2}(1+2\alpha\varepsilon)^{-1}d\varepsilon}{\int_{0}^{\infty} (-\frac{\partial f}{\partial\varepsilon})\tau_{total}\varepsilon^{3/2}(1+\alpha\varepsilon)^{3/2}(1+2\alpha\varepsilon)^{-1}d\varepsilon}-(\frac{\int_{0}^{\infty} (-\frac{\partial f}{\partial\varepsilon})\tau_{total}\varepsilon^{5/2}(1+\alpha\varepsilon)^{3/2}(1+2\alpha\varepsilon)^{-1}d\varepsilon}{\int_{0}^{\infty} (-\frac{\partial f}{\partial\varepsilon})\tau_{total}\varepsilon^{3/2}(1+\alpha\varepsilon)^{3/2}(1+2\alpha\varepsilon)^{-1}d\varepsilon})^{2}]$The carrier concentration *n*:

The drift mobility can be expressed by:

$$\mu=\frac{e}{m_{I}^{*}}\frac{\int_{0}^{\infty} (-\frac{\partial f}{\partial\varepsilon})\tau_{total}(1+\alpha\varepsilon)^{3/2}(1+2\alpha\varepsilon)^{-1}d\varepsilon}{\int_{0}^{\infty} (-\frac{\partial f}{\partial\varepsilon})(\varepsilon+\alpha\varepsilon^{2})^{3/2}d\varepsilon}$$

Here, $m_{I}^{*}$ is the inertial effective mass and can be calculated by $m_{I}^{*}=\frac{3m_{d}^{*}}{N_{\text{V}}^{2/3}(2K^{1/3}+K^{-2/3})}$, *K* is the anisotropy factor of effective mass of the carrier pocket along the two directions. *K*=10 was adopted here.

**Reference:**

(1) Schmetterer, C.; Flandorfer, H.; Richter, K. W.; Saeed, U.; Kauffman, M.; Roussel, P.; Ipser, H. A new investigation of the system Ni–Sn. *Intermetallics* **2007**, *15*, 869.

(2) Berche, A.; Tédenac, J. C.; Jund, P. Phase stability of nickel and zirconium stannides. *Journal of Physics and Chemistry of Solids* **2017**, *103*, 40.

(3) Subasic, N. Thermodynamic evaluation o Sn-Zr phase diagram. *Calphud.*, *22*, 157.
